# Two Fast GC-MS Methods for the Measurement of Nicotine, Propylene Glycol, Vegetable Glycol, Ethylmaltol, Diacetyl, and Acetylpropionyl in Refill Liquids for E-Cigarettes

**DOI:** 10.3390/molecules28041902

**Published:** 2023-02-16

**Authors:** Ioanna Dagla, Evagelos Gikas, Anthony Tsarbopoulos

**Affiliations:** 1 Bioanalytical Laboratory, GAIA Research Center, The Goulandris Natural History Museum, 14562 Kifissia, Greece; 2 Laboratory of Analytical Chemistry, Faculty of Chemistry, National and Kapodistrian University of Athens, Panepistimioupolis Zografou, 15784 Athens, Greece; 3 Department of Pharmacology, Medical School, National and Kapodistrian University of Athens, 11527 Athens, Greece

**Keywords:** GC-MS, nicotine, ethylmaltol, diacetyl, propylene glycol, quality control procedure, e-liquids, e-cigarettes

## Abstract

The use of e-cigarettes (ECs) has become increasingly popular worldwide, even though scientific results have not established their safety. Diacetyl (DA) and acetylpropionyl (AP), which can be present in ECs, are linked with lung diseases. Ethyl maltol (EM)—the most commonly used flavoring agent—can be present in toxic concentrations. Until now, there is no methodology for the determination of nicotine, propylene glycol (PG), vegetable glycerin (VG), EM, DA, and acetylpropionyl in e-liquids that can be used as a quality control procedure. Herein, gas chromatography coupled with mass spectrometry (GC-MS) was applied for the development of analytical methodologies for these substances. Two GC-MS methodologies were developed and fully validated, fulfilling the standards for the integration in a routine quality control procedure by manufacturers. As proof of applicability, the methodology was applied for the analysis of several e-liquids. Differences were observed between the labeled and the experimental levels of PG, VG, and nicotine. Three samples contained EM at higher concentrations compared to the other samples, while only one contained DA. These validated methodologies can be used for the quality control analysis of EC liquid samples regarding nicotine, PG, and VG amounts, as well as for the measurement of the EM.

## 1. Introduction

Electronic cigarettes (ECs) are nicotine-delivery products that, instead of tobacco, contain a solution of nicotine benzoate salt in a propylene glycol (PG) and glycerol (vegetable glycerin, VG) base and various flavoring agents. The base is made from PG, VG, or a mixture of the two in various ratios, diluted in purified water. Nicotine concentration varies from 0 mg/mL to 18 mg/mL. The wide variety of tastes (e.g., sweet, cool, bitter, harsh) contributes to a higher likability of EC products and higher initiation rates of vaping [1]. According to the “E-cigarette and Vape Market Size Report, 2022–2030” (https://www.grandviewresearch.com/industry-analysis/e-cigarette-vaping-market, accessed on 27 January 2023), “*The global e-cigarette and vape market size was valued at USD 18.13 billion in 2021 and is expected to expand at a compound annual growth rate (CAGR) of 30.0% from 2022 to 2030*”. The Center for Disease Control and Prevention states that “*about 1 in 5 high school students and 1 in 20 middle school students reported using e-cigarettes in 2020*” (https://www.cdc.gov/tobacco/features/back-to-school/index.html, accessed on 27 January 2023).

Although ECs are likely to be far less harmful than conventional cigarettes, they can be correlated with several health hazards [2]. Concerns have been raised about the potential inhalation toxicity of the flavoring chemicals that are added to ECs to create flavors [3]. Cinnamaldehyde, benzaldehyde, ethyl vanillin, ethyl maltol (EM), and vanillin are specific chemicals that have been linked to cytotoxic effects on respiratory cells [4]. The potential health risk is challenging since the concentration of flavoral chemicals is not known. Manufacturers are not obliged to report the chemical substances or concentrations, and the FDA regulations do not propose guidelines for EC ingredients.

For the determination of nicotine and flavoring chemical concentrations in e-liquid refill samples, a limited number of analytical methodologies exists based on gas chromatography–mass spectrometry (GC-MS) [5,6,7,8,9,10], liquid chromatography–mass spectrometry (LC-MS) [11], and nuclear magnetic resonance (NMR) [12,13].

A systematic review of refillable e-liquid samples demonstrated that the actual concentration of nicotine might vary considerably from labeled concentrations [14]. Among the flavoring agents, EM, which has a cotton candy fragrance, is the most common component in EC liquids [11]. A study demonstrated that EM was contained in 80% of the tested e-liquids at concentrations 100 times its cytotoxic concentration [15]. The concentration of EM ranges from 0.001 to 10 mg/mL in e-liquid refills [10]. Based on the IC_50_ data, EM is the most toxic ingredient among the flavoring agents, and more interestingly, it has been shown that the cytotoxicity of refill fluids is directly correlated with EM concentrations in the fluids [10]. Furthermore, the cytotoxicity of produced aerosols during vaping has been strongly correlated with nicotine and EM concentrations [9]. EM promotes free radicals in aerosols in a concentration-dependent manner that cause damage to proliferation, survival, and inflammation pathways in the cell [7]. This fact emphasizes the indispensable need for regulations regarding the flavoring chemicals in e-liquids. Diacetyl (DA) is also an ingredient used in e-liquids for its characteristic butter flavor note and was found in more than 60% of samples [16]. Moreover, the formation of DA could be observed during aerosol generation from e-liquids [17]. Unfortunately, DA has been linked to the development of obliterative bronchiolitis, which is an irreversible, life-threatening lung disease [17,18,19,20]. In addition, DA has been associated with Alzheimer’s disease, as it has been demonstrated to aggregate amyloid-β [21]. Flavoring alternatives to DA have been used in the food industry, e.g., 2,3-pentane-dione, 2,3-heptanedione, and acetoin, but these chemicals cause respiratory hazards as well [16]. FDA suggests that the presence of specific constituents, including DA and acetylpropionyl (AP), should be considered in e-liquids and aerosols to characterize that a product is “appropriate for the protection of public health” [17]. A few analytical methodologies have been developed for the analysis of these chemicals using gas chromatography–electron capture detector [16], GC-MS [22], UPLC-MS [17], and HPLC-UV [23].

Herein, two fast analytical methodologies have been developed for the analysis of nicotine, PG, VG, EM, DA, and AP. These methodologies can be used for the quality control analysis of e-liquids assessing the nicotine, PG, and VG amounts, as well as for the measurement of the toxic flavor chemical EM. The latter (EM concentration) has been directly correlated with the cytotoxicity of e-liquids. The presence of DA and AP can be estimated in a quick analytical procedure. Several methodologies have been previously reported for the quantitation of these substances in e-liquids, but herein, we present a two-stage methodology for the simultaneous determination of PG, VG, nicotine, and EM and the estimation of DA and AP levels. Until now, the reported methodologies for the determination of the PG and VG levels have included NMR technology [24], GC-MS methodologies with long total run time [25,26], and an SPME preparation step prior to analysis [5]. This is the first methodology for the analysis of the combination of these substances using GC-MS. The presented methodology is fast and can be used as a quality control method employing only the GC-MS instrumentation, which is common and familiar to many industries.

## 2. Results and Discussion

### 2.1. Method Development

Nicotine, PG, VG, and EM were selected for screening during the quality control of e-liquids. Furthermore, the examination for the potential presence of DA and AP in e-liquids was deemed essential, as these substances are very toxic. Prior to the GC-MS analysis, a derivatization step was necessary. Two derivatization reagents were selected, owing to the different structures of the substances. The analysis of the hydroxy groups of PG, VG, and EM was based on the derivatization with the BSTFA + TMCS reagent (first method), while the analysis of carbonyl groups of DA and AP was performed with derivatization using the o-phenylenediamine (second method). Nicotine was not derivatized.

**Figure 1:**
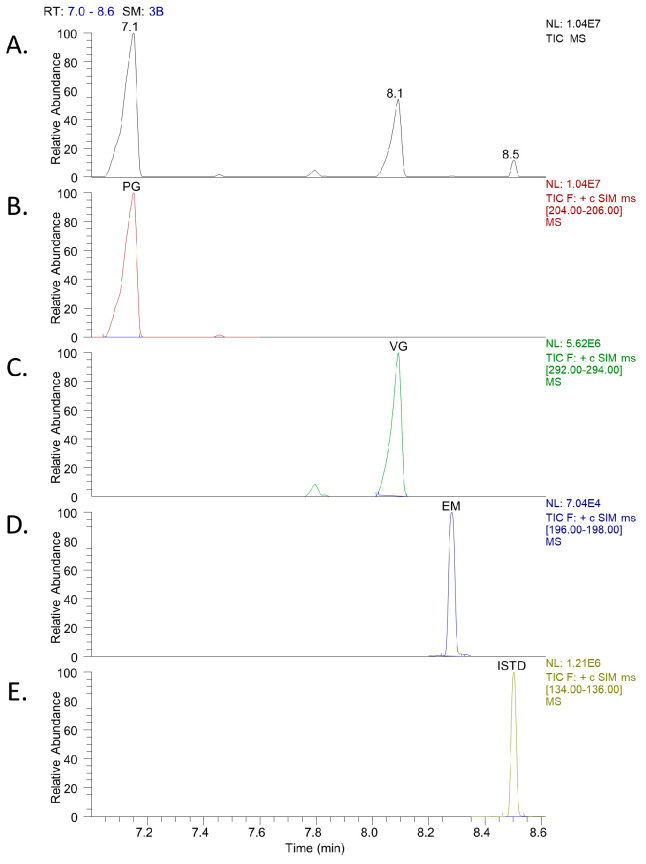
(**A**). Total ion current chromatogram of PG, VG, EM, and ISTD using GC-MS. (**B**–**E**). Extracted ion chromatograms of PG (*m*/*z* 205), VG (*m*/*z* 293), EM (*m*/*z* 197), and ISTD (*m*/*z* 135).

For the first method, the calibration points of EM, PG, and VG were combined, thus shortening the analysis time. A typical chromatogram of PG, VG, EM, and ISTD is presented in Figure 1.

To ensure that the concentration of EM was not affected by the different matrices (different ratios of PG/VG in each calibration point), EM at a concentration of 0.3 mg/mL was spiked in the tested ratios of PG/VG (100/0, 80/20, 70/30, 50/50, 30/70, and 0/100), and the samples were analyzed according to the described methodology. The %RSD of the EM in the examined samples (*n* = 6) was 1.3%, indicating that the ratio of PG/VG did not affect the dilution of EM. Therefore, the combination of calibration points did not result in fault results. Furthermore, the proposed methodology did not require prior knowledge of the PG/VG ratio in e-liquids for the analysis of EM. A blank chromatogram after the addition of derivatization agents and the ISTD is given in Figure S1 (Supplementary Material).

**Figure 2:**
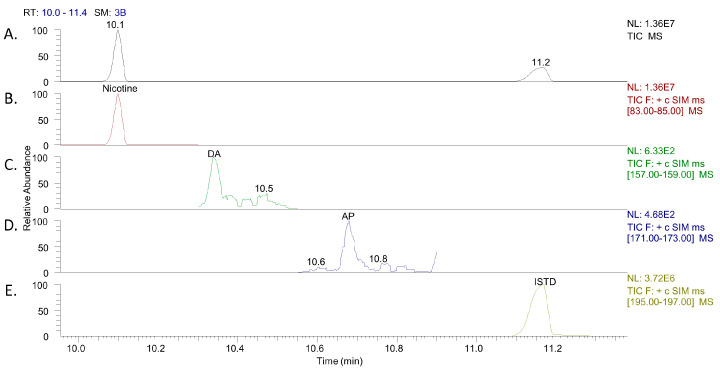
(**A**). Total ion current chromatogram of nicotine, DA, AP, and ISTD using GC-MS. (**B**–**E**). Extracted ion chromatograms of nicotine (*m*/*z* 84), 5 μg/mL DA (*m*/*z* 158), 5 μg/mL AP (*m*/*z* 172), and ISTD (*m*/*z* 196).

For the second method, the PG/VG ratio of 50/50 was selected as the matrix for the calibration curve of nicotine. The %RSD of nicotine (9 mg/mL, *n* = 6) was 2.1% in different ratios of PG/VG; thus, one ratio of PG/VG was selected to simplify the methodology. For DA and AP, no calibration curves were constructed. Taking into consideration that the presence of those substances in e-liquids was not desirable, it was deemed that the measurement of their level was beyond the limits of a quality control methodology. The described methodology defines a threshold level of 5 μg/mL to show if an e-liquid contains DA and AP. The dilution of DA and AP in PG/VG was not affected by the ratio of the matrix (%RSD = 1.8, *n* = 6). A typical chromatogram of nicotine, DA, and AP is presented in Figure 2. A blank chromatogram after the addition of derivatization agents and the ISTD is given in Figure S2.

### 2.2. Method Validation

#### 2.2.1. Selectivity and Specificity

In both methodologies, it was observed that the matrices (derivatization reagents) did not interfere with the detection of the target analytes. The specificity of the current methodologies was ensured by monitoring the specific ions of nicotine, EM, PG, VG, DA, and AP.

#### 2.2.2. Fitted Models

The calibration curves were constructed for the range of 0–10 mL/mL for PG and VG, 2–20 mg/mL for nicotine, and 0.1–0.5 mg/mL for EM. The areas of targeted compounds were divided by the area of ISTD. The linear and quadratic models were examined. The correlation coefficient (R^2^) was better in the quadratic models than in the linear models for all the substances. The percent errors of the back-calculated values were compared for the two models. That error was calculated by the following equation:

$$\%E=\frac{C_{theoretical}-C_{experimental}}{C_{theoretical}}\times 100$$

where *C_theoretical_* is the theoretical concentration level of each substance and *C_experimental_* is the concentration calculated by the linear or the quadratic regression. The back-calculated values using the quadratic regression presented lower percent errors than those obtained with the linear equation. Generally, the absolute average %E of the back-calculated values with the quadratic and linear regressions were 2.2% and 6.4%, respectively, for nicotine; 2.2% and 10.6% for PG; 2.5% and 4.6% for VG; and 0.1% and 2.2% for EM. Therefore, the calibration curves were established by applying the quadratic regression. The best-fit values of the intercept (B0), the coefficient of the linear term (B1), and the coefficient of the squared term (B2), as well as the standard errors associated with the coefficients, the R^2^ and the standard error of estimate (Sy.x), are presented in Table 1. Even though the B2 term has a high standard deviation for some analytes (e.g., PG), the quadratic model was selected over the linear.

#### 2.2.3. Accuracy and Precision

The intra-day and inter-day accuracy expressed as percent standard error from the nominal value (%E) was assessed by analyzing samples at three concentration levels and at three analytical runs. All the models exhibited accuracy lower than 7.5% at the three tested levels. Repeatability (the precision under the same operating condition over a short interval of time) and intermediate precision (the variations between different analytical days, *n* = 3) were expressed as percent relative standard deviation (%RSD). The results are presented in Table 2.

#### 2.2.4. Stability and Robustness

Stability was examined at the same levels by injecting the same sample (*n* = 3) every three hours (autosampler stability). The results showed that all of the tested compounds were stable at the duration of the data acquisition. The robustness was examined by making deliberate changes (±5%) in the GC parameters (injector temperature and carrier gas flow rate).

#### 2.2.5. Carry-over

Injections of the upper limit of quantitation (ULOQ) were performed. No carry-over effect was observed since a non-detectable amount of the analytes was found in the blank injected samples.

#### 2.2.6. Screening and Quantification of E-Liquid Samples

The validated methodologies were used for the determination of nicotine, EM, PG, VG, DA, and AP in a subset of e-liquid samples. The concentrations of the substances are expressed as % *w*/*v* (which is equivalent to mg/100 mL concentration), and the results are presented in Table 3. Apart from sample_1, none of the samples was detected positive for DA (LOD = 5 μg/mL). No other sample was found positive for DA and AP. In most of the tested e-liquids, the results revealed that the levels of PG and VG agreed with those claimed by the manufacturer (<±10.0%Ε). However, some samples presented >±10.0%E for PG and VG (for example, sample_8 and sample_22). In most cases, the amount of nicotine determined by the developed methodology was about the same as that claimed by the manufacturer (<27.8% E), except for the sample_25 that presented 41.7%E from the labeled value. The amount of EM was not stated in the e-liquid labels; thus, the %E could not be calculated.

Three samples (Sample_5, Sample _15, and Sample_20) contained EM at higher concentrations compared to the other samples. In fact, those levels of EM were above the higher point of the calibration curve. The analysis of those samples was performed via the appropriate dilution of the sample so that the concentration was within the calibration range. In the literature, there is a lack of information on the safe limit of EM concentration in e-liquids. The study of Omaiye et al. claimed that 46% of the tested e-liquid samples contained EM (0.008–3.13%) in concentrations higher than those added to edible products (up to 0.0142%) and in final products of soap (up to 0.06%), detergents (up to 0.006%), and creams and lotions (up to 0.01%) [15]. The oral LD50 of EM is 1150 mg/Kg for rats, but there is no information on the safe intake in humans. Thus, there is an imperative need to establish the maximum allowed nontoxic concentration for EM in e-liquids.

## 3. Materials and Methods

### 3.1. Chemicals and Reagents

Methanol ≥99.9% was purchased from Thermo Fisher Scientific (Waltham, MA, USA), and acetone was from Carlo Erba Reagents (Val de Reuil CEDEX, France). EM, 2,3-Butanedione 97% (DA), 2,3-Pentanedione 97% (AP), N,OBis(trimethylsilyl)trifluorocetamide (BSTFA) 1%–TMCS 99%, and 3-methoxyphenethyl alcohol (internal standard—ISTD) were purchased from Sigma-Aldrich (Steinheim, Germany). The reagent o-Phenylenediamine 98% was purchased from Thermo Fisher Scientific (Waltham, MA, USA). Nicotine, propylene glycol (PG), and vegetable glycol (VG) were provided by NOBACCO (Koropi, Greece). Ultrapure water was produced by a Millipore Direct-Q System (Molsheim, France).

### 3.2. E-Liquid Samples

Twenty-six e-liquid samples provided by NOBACCO were intended to be screened for nicotine, EM, PG/VG ratio, and the presence of DA and AP. All samples were stored at ambient temperature and protected from light.

### 3.3. Preparation of Standard Solutions

First method: A stock solution of EM was prepared in methanol at a concentration of 10 mg/mL and was stored at –30 °C to avoid sample degradation. Stock solutions of PG and VG were prepared at ratios of PG/VG—80/20, 70/30, 50/50, and 30/70 mL/mL.

The calibration solutions were obtained by diluting the EM stock solution with 1 mL of PG/VG to the concentration levels of 0.1, 0.2, 0.3, 0.4, and 0.5 mg/mL. Each addition of EM was performed in different ratios of PG/VG so that three calibration curves were obtained in one analytical run. The amounts of EM (mg/mL), PG (mL), and VG (mL) in calibration points (CP) were 1st CP: 0.1, 100, 0; 2nd CP: 0.2, 80, 20; 3rd CP: 0.3, 70, 30; 4th CP: 0.4, 50, 50; 5th CP: 0.5, 30, 70; and 6th CP: 0.5, 0, 100, respectively. The ISTD was added to each sample at a final concentration level of 6 mg/mL.

Derivatization was performed by adding 40 μL of BSTFA + TMCS to 1 μL of the sample. Finally, the solutions were thoroughly mixed and maintained at 55 °C for 30 min.

Second method: Stock solutions of DA and AP were prepared in acetone at concentrations of 1 mg/mL. A stock solution of o-phenylenediamine was prepared in methanol at a concentration of 1 mg/mL.

Calibration standards for nicotine were prepared in PG/VG 50/50 mL/mL at concentration levels of 2, 3, 6, 9, 12, 18, and 20 mg/mL. DA and AP were added to each sample at concentration levels of 5 μg/mL. The ISTD was added to each sample at concentration levels of 6 mg/mL.

Derivatization was performed by adding 20 μL of o-phenylenediamine to 5 μL of the sample. Finally, the solutions were thoroughly mixed and maintained at room temperature for 5 min.

### 3.4. Sample Preparation

A total of 1 mL of each e-liquid was transferred in 2 mL Eppendorf tube, 6 μL of ISTD were added, and the solution was vortexed for 30 s.

For the measurement of EM, PG, and VG, 40 μL of BSTFA + TMCS were added to 1 μL of the sample, and the solution was maintained at 55 °C for 30 min.

For the measurement of nicotine, DA, and AP, 20 μL of o-phenylenediamine was added to 5 μL of the sample, and the solution was maintained at room temperature for 5 min.

### 3.5. Instrumentation

The Thermo Trace 2000 series GC (ThermoQuest, Waltham, MA, USA) system coupled with the Q plus (ThermoQuest, Waltham, MA, USA) mass spectrometer was used. The system was equipped with the AS 2000 (ThermoQuest, Waltham, MA, USA) autosampler. Analysis was performed using the Xcalibur version 1.2 (Thermo Fisher Scientific, Waltham, MA, USA) software. An SGE fused silica capillary column BP-5 ms (30 m × 0.25 mm × I.D. 0.25 μm film thickness) (Trajan Scientific and Medical, Victoria, Australia) was used for the chromatographic separation. Helium was used as the carrier gas at a constant flow rate of 0.8 mL/min. The injections were in split mode with a 1:5 ratio, and the injector temperature was 250 °C. The ionization of the compounds was carried out by EI in the positive ion mode at an electron energy of 70 eV with a source temperature of 230 °C, whereas the ion trap temperature was 75 °C. The injection volume was 1 μL. The oven temperature was 50 °C (held for 5 min), programmed to reach 310 at 30 °C/min in the first method and at 60 °C/min in the second method. The final temperature was kept for 1 min. The selected ion monitoring (SIM) mode was selected. In the first method, the ions 205 *m*/*z*, 293 *m*/*z*, 197 *m*/*z*, and 135 *m*/*z* were selected for the detection of PG, VG, EM, and ISTD, respectively. In the second method, the ions 84 *m*/*z*, 158 *m*/*z*, 172 *m*/*z*, and 196 *m*/*z* were selected for the detection of nicotine, DA, AP, and ISTD, respectively.

### 3.6. Method Validation

The method was validated by examining linearity, precision (repeatability and intermediate precision), accuracy, reproducibility, stability, robustness, the limit of detection (LOD), and the limit of quantitation (LOQ) according to the ICH Q2(R1) analytical procedure guidelines (https://www.ema.europa.eu/en/documents/scientific-guideline/ich-q-2-r1-validation-analytical-procedures-text-methodology-step-5_en.pdf, accessed on 30 November 2022).

## 4. Conclusions

A two-stage analytical methodology was developed for the simultaneous determination of nicotine, PG, VG, EM, DA, and AP. The importance of this analytical methodology depends on the fact that DA and AP are referred to as harmful and potentially harmful constituents (HPHCs) of e-liquids, and electronic cigarettes are not totally safe for human health.

The validated methodology is fast and can be applied for the quality control of e-liquids by manufacturers, as it is based on the familiar and commonly available GC-MS instrumentation. As proof of applicability, the validated methods were successfully applied on a small set of EC liquid samples, indicating that this methodology could be used for routine quality control analyses of EC liquids. The quality control of e-liquids is an essential procedure as differences of actual against the labeled concentrations of the PG, VG, and nicotine have been shown. Another advantage of the developed, fast methodology is the possibility for quantitative determination of one of the most used and toxic flavor chemicals, EM, as well as the estimation of toxic DA and AP levels. The analysis of EM, DA, and AP is especially important, as the maximum allowable levels compared to the proposed occupational exposure limits are still under scientific examination.

Finally, the suggested methodology, apart from a quality control routine procedure, can be applied for the examination of the stability of e-liquids under different conditions of storage, as many of the users of these e-liquid refill samples keep them, for instance, at their homes or offices in the direct sunlight. Furthermore, the e-liquid inside the vaporizer undergoes repeated cycles of temperature variations (from vaporing point to room temperature) during vaping, thus making the assessment of the e-liquid content imperative for safeguarding the quality of the refill liquids.

Part of this work was presented at the 2nd Scientific Summit on Tobacco Harm Reduction (Greece, Athens, 29–30 May 2019).

## Figures and Tables

**Table 1:** The best-fit values B0, B1, and B2, the standard errors associated with the coefficients, the correlation coefficient (R^2^), and the standard error of the estimate (Sy.x) of the quadratic models for PG, VG, EM, and nicotine.

Compounds	Best-fit Values			Std. Error			R^2^	Sy.x
	B0	B1	B2	B0	B1	B2		
PG	0.04949	8.299	–0.06763	0.04882	0.3841	0.04899	0.9994	0.2195
VG	0.2189	0.1991	0.000476	0.3863	0.0183	0.000173	0.9990	0.4298
EM	–0.02828	0.7924	–0.2316	0.01217	0.09273	0.1516	0.9990	0.005673
Nicotine	0.000615	0.2408	–0.00391	0.06438	0.01515	0.000681	0.998	0.05609

**Table 2:** Accuracy (intra-day and inter-day), repeatability, intermediate precision, stability, and robustness assessed for PG, VG, EM, nicotine, DA, and AP.

Substance (Levels)	Accuracy (%E, *n* = 3)		Precision (%RSD, *n* = 3)		Stability (%RSD, *n* = 3)	Robustness (%RSD, *n* = 3)
	Intra-day	Inter-day	Repeatability	Intermediate Precision		
PG (mL/10 mL)						
2	1.05	5.35	2.7	1.1	2.4	
5	−5.63	−7.53	7.3	2.8	5.2	<1.2
10	2.55	3.05	3.0	2.5	3.2	
VG (mL/10 mL)						
2	5.47	2.36	3.4	4.3	2.5	
5	2.51	–5.2	4.1	1.2	5.0	<2.0
10	6.24	2.84	0.3	3.5	2.9	
EM (mg/mL)						
0.1	2.1	0.05	1.7	1.9	1.5	
0.3	2.79	2.34	2.3	1.8	1.8	<1.3
0.5	−2.51	−1.58	3.4	3.5	2.1	
Nicotine (mg/mL)						
3	0.02	3.15	0.7	0.0	1.0	
12	3.66	7.53	5.3	1.7	1.6	<1.27
20	−1.95	−5	5.0	1.5	1.2	
DA (μg/mL)						
5	1.23	2.65	2.4	4.5	2.6	<2.4
AP (μg/mL)						
5	−0.59	2.36	1.3	1.1	1.6	<1.9

**Table 3:** The levels (expressed as % *w*/*v*) of PG, VG, nicotine, and EM in the tested e-liquids calculated by the developed GC-MS methodology. The amounts of PG, VG, and nicotine claimed by the manufacturer are also presented. The error (%E) of the calculated (GC-MS–derived) vs. the claimed values is given in parenthesis. The calculation for the %E was based on the mathematical formula $\%E=\frac{C_{claimed}-C_{calcd.}}{C_{claimed}}\times 100$.

Samples	PG % *w*/*v*		VG % *w*/*v*		Nicotine % *w*/*v*		EM % *w*/*v*
	Claimed	Calcd. (%E)	Claimed	Calcd. (%E)	Claimed	Calcd. (%E)	Calcd.
Sample_1	70	68.6 (2.0)	30	29.6 (1.2)	1.8	1.7 (5.6)	0.003
Sample_2	70	67.7 (3.2)	30	31.6 (–5.4)	0.6	0.6 (0.0)	0.004
Sample_3	70	75.0 (–7.1)	30	25.0 (16.8)	0.0	0.0 (0.0)	0.001
Sample_4	70	64.4 (8.0)	30	34.4 (–14.8)	1.2	1.1 (8.3)	0.002
Sample_5	60	55.2 (8.0)	40	42.8 (–7.1)	1.8	1.8 (0.0)	0.171
Sample_6	60	56.6 (5.7)	40	41.6 (–4.0)	1.8	1.8 (0.0)	0.006
Sample_7	60	56.5 (5.9)	40	42.4 (–5.9)	1.2	1.2 (0.0)	0.02
Sample_8	60	70.1 (–16.8)	40	29.9 (25.4)	0.0	0.0 (0.0)	0.003
Sample_9	70	69.6 (0.5)	30	29.3 (2.4)	1.2	1.1 (8.3)	0.002
Sample_10	70	70.5 (–0.7)	30	28.9 (3.8)	0.6	0.6 (0.0)	0.006
Sample_11	50	53.0 (–6.0)	50	45.7 (8.6)	1.2	1.3 (–8.3)	0.012
Sample_12	50	46.5 (6.9)	50	53.0 (–5.9)	0.6	0.5 (16.7)	0.000
Sample_13	50	50.5 (–0.9)	50	49.2 (1.5)	0.3	0.3 (0.0)	0.009
Sample_14	50	48.4 (3.2)	50	51.3 (–2.6)	0.3	0.3 (0.0)	0.000
Sample_15	50	47.2 (5.6)	50	52.1 (–4.3)	0.6	0.6 (0.0)	0.11
Sample_16	70	71.7 (–2.5)	30	27.0 (9.9)	1.2	1.2 (0.0)	0.01
Sample_17	50	47.7 (4.7)	50	51.1 (–2.1)	1.2	1.3 (–8.3)	0.000
Sample_18	0	7.8 (0.0)	100	91.7 (8.3)	0.6	0.5 (16.7)	0.013
Sample_19	70	65.5 (6.4)	30	33.0 (–9.9)	1.8	1.5 (16.7)	0.000
Sample_20	70	74.5 (–6.5)	30	23.9 (20.2)	1.8	1.3 (27.8)	0.18
Sample_21	100	100.0 (0.0)	0	0.0 (0.0)	0.0	0.0 (0.0)	0.000
Sample_22	70	81.7 (–16.6)	30	17.0 (43.4)	1.2	1.4 (–16.7)	0.000
Sample_23	70	66.6 (4.8)	30	31.3 (–4.4)	1.8	2.0 (–11.1)	0.000
Sample_24	0	0.0 (0.0)	100	94.2 (5.8)	0.6	0.6 (0)	0.000
Sample_25	50	44.6 (10.9)	50	53.7 (–7.4)	1.2	1.7 (–41.7)	0.000
Sample_26	50	45.0 (9.9)	50	54.3 (–8.5)	0.6	0.7 (–16.7)	0.000

## Data Availability

The data presented in this study are available in this article and supplementary material.

## Supplementary Materials

The following supporting information is available online at https://www.mdpi.com/article/10.3390/molecules28041902/s1, Figure S1: Blank chromatogram after addition of derivatization agent and ISTD acquired using the GC-MS methodology developed for the measurement of PG, VG, and EM, Figure S2: Blank chromatogram after addition of derivatization agent and ISTD acquired using the GC-MS methodology developed for the measurement of nicotine, DA, and AP.

## Sample Availability

Samples of the compounds are not available from the authors.
