# Supplementary material for: Two Fast GC-MS Methods for the Measurement of Nicotine, Propylene Glycol, Vegetable Glycol, Ethylmaltol, Diacetyl, and Acetylpropionyl in Refill Liquids for E-Cigarettes
Source: Molecules. 2023 Feb 16;28(4):1902. doi: 10.3390/molecules28041902 (PMC9961753; doi:10.3390/molecules28041902)
Supplement: Supplementary file 1 [file molecules-28-01902-s001.zip › molecules-2227231-supplementary.pdf]

Supplementary Material

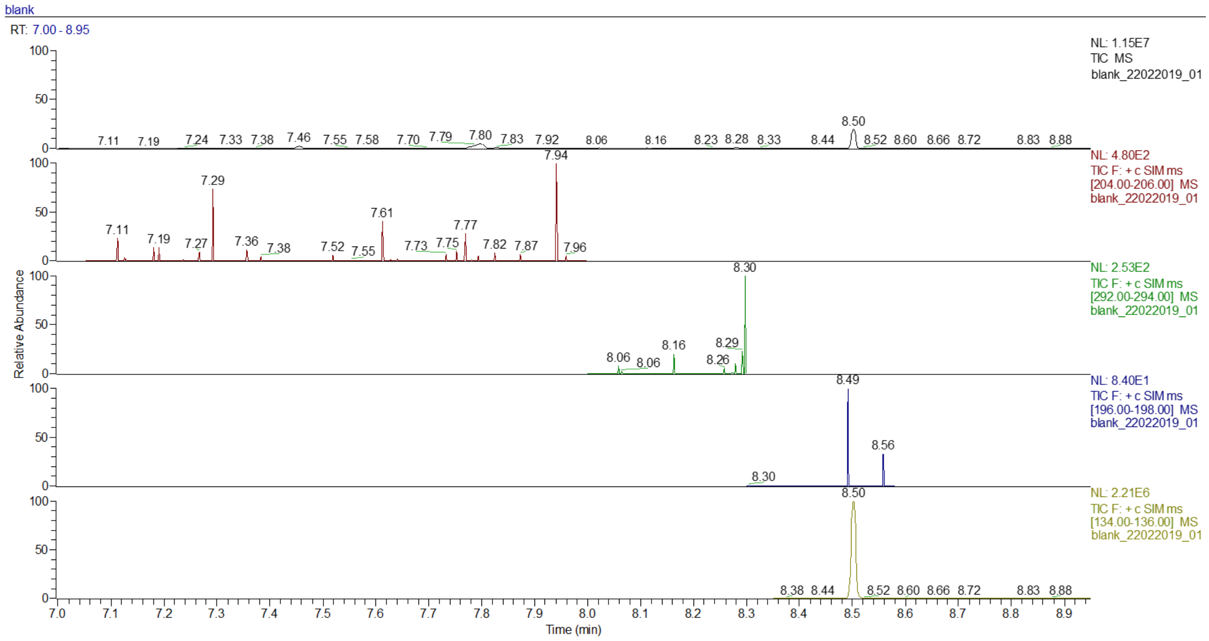

Figure S1. Blank chromatogram after addition of derivatization agent and ISTD acquired using the GC-MS methodology developed for the measurement of PG, VG and EM.

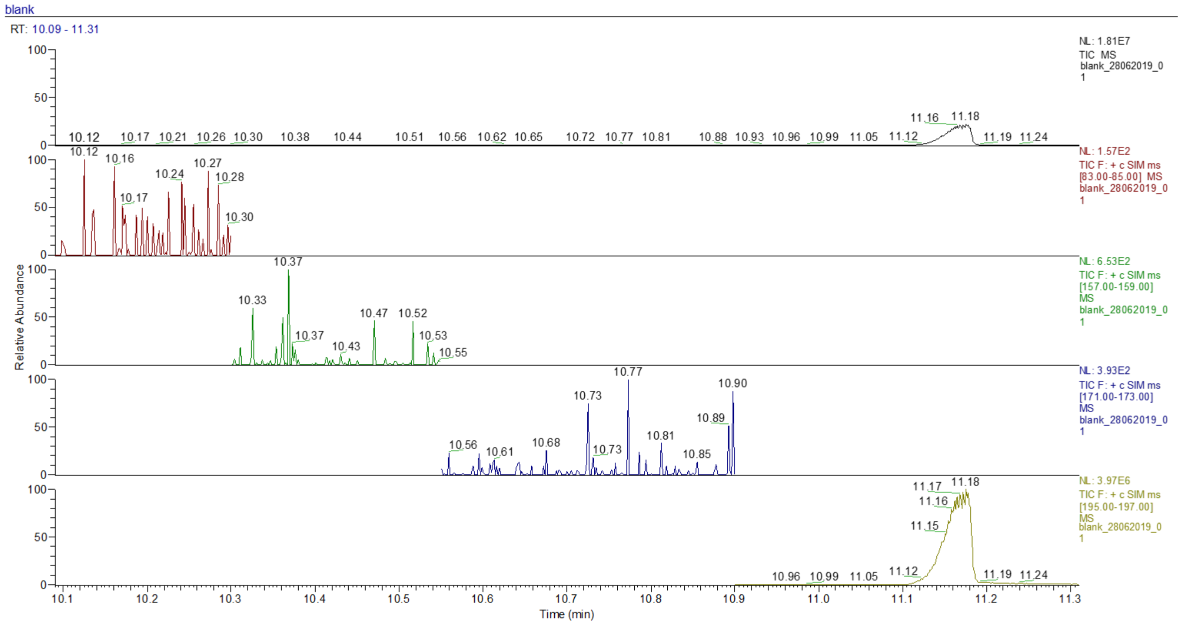

Figure S2. Blank chromatogram after addition of derivatization agent and ISTD acquired using the GC-MS methodology developed for the measurement of nicotine, DA and AP.
